# Effects of baseline heart rate at sea level on cardiac responses to high-altitude exposure

**DOI:** 10.1007/s10554-020-01769-w

**Published:** 2020-01-17

**Authors:** Jingdu Tian, Chuan Liu, Yuanqi Yang, Shiyong Yu, Jie Yang, Jihang Zhang, Xiaohan Ding, Chen Zhang, Rongsheng Rao, Xiaohui Zhao, Lan Huang

**Affiliations:** 1 Institute of Cardiovascular Diseases of PLA, the Second Affiliated Hospital, Third Military Medical University (Army Medical University), Chongqing, 400037 China; 2 Department of Cardiology, the Second Affiliated Hospital, Third Military Medical University (Army Medical University), 183 Xinqiao street, Shapingba district, Chongqing, 400037 China; 3 Department of Geriatric Cardiology, Chinese PLA General Hospital, Beijing, 100038 China; 4 Department of Medical Ultrasonics, the Second Affiliated Hospital, Third Military Medical University (Army Medical University), Chongqing, 400037 China

**Keywords:** High altitude, Cardiac function, Heart rate, Echocardiography

## Abstract

High-altitude (HA) exposure has been widely considered as a cardiac stress, and associated with altered cardiac function. However, the characteristics of cardiac responses to HA exposure are unclear. In total, 240 healthy men were enrolled and ascended to 4100 m by bus within 7 days. Standard echocardiography and color tissue Doppler imaging were performed at sea level and at 4100 m. In all subjects, HA exposure increased HR [65 (59, 71) vs. 72 (63, 80) beats/min, p < 0.001] but decreased the stroke volume index (SVi) [35.5 (30.5, 42.3) vs. 32.9 (27.4, 39.5) ml/m^2^, p < 0.001], leading to an unchanged cardiac index (CI). Moreover, baseline HR was negatively correlated with HA exposure-induced changes in HR (r = − 0.410, p < 0.001) and CI (r = − 0.314, p < 0.001). Following HA exposure, subjects with lowest tertile of baseline HR showed an increased HR [56 (53, 58) vs. 65 (58, 73) beats/min, p < 0.001], left ventricular ejection fraction (LVEF) [61.7 (56.5, 68.0) vs. 66.1 (60.7, 71.5) %, p = 0.004] and mitral S′ velocity [5.8 ± 1.4 vs. 6.5 ± 1.9 cm/s, p = 0.040]. However, subjects with highest tertile of baseline HR showed an unchanged HR, LVEF and mitral S′ velocity, but a decreased E′ velocity [9.2 ± 2.0 vs. 8.4 ± 1.8 cm/s, p = 0.003]. Our findings indicate that baseline HR at sea level could determine cardiac responses to HA exposure; these responses were characterized by enhanced LV function in subjects with a low baseline HR and by reduced LV myocardial velocity in early diastole in subjects with a high baseline HR.

**Electronic supplementary material:**

The online version of this article (10.1007/s10554-020-01769-w) contains supplementary material, which is available to authorized users.

## Introduction

High-altitude (HA) exposure has been widely considered as a cardiac stress during short-term or prolonged periods at HA for the purposes of recreation, sports, work or military training. Well-adapted cardiac performance may facilitate oxygen delivery to tissues when no oxygen therapy is available. It has been well documented that the global systolic left ventricular (LV) and right ventricular (RV) functions are well preserved, but both ventricles show altered filling patterns, subendocardial systolic dysfunction, and the increase in mean pulmonary arterial pressure (mPAP) [1–3]. Notably, there may be a potential association between altered cardiac functions and the incidence of acute mountain sickness [4], whereas the increase in mPAP may limit exercise capacity at HA [5, 6]. Although the cardiac responses to HA exposure are of great significance, the detailed characterizations are largely unknown.

The initial cardiac responses to acute HA exposure include the increase in heart rate (HR) and cardiac output (CO) but with no change in stroke volume (SV). However, after a few days of acclimatization (3–5 days), the CO begins to decrease and returns to baseline level with a higher HR and a lower SV [7]. The increase in HR is mainly due to sympathetic activation [8], whereas the decrease in SV is attributed to a decrease in blood volume, an increase in mPAP, the impairment of myocardial relaxation and an increase in HR, all of which contribute to the altered filling patterns of both ventricles [9]. During these physiological processes, the change in HR seems to be a critical determinant of cardiac performance at HA. In addition, evidence has shown that the resting HR is a predictor of LV dysfunction and heart failure, and a high HR may contribute to the development of regional and global LV dysfunction [10]. Furthermore, HR may directly impact cardiac function, and a low HR might be more beneficial for patients with systolic heart failure than an intermediate HR [11]. However, whether the baseline HR of lowlanders exhibits diverse cardiac responses during HA travels is still unclear.

Considering that tissue Doppler imaging (TDI) allows for the noninvasive determination of myocardial velocity in a shape-independent manner and may provide an exact evaluation for cardiac function [12], we conducted the present study to investigate the cardiac responses under acute HA exposure and the impact of baseline HR by using standard Doppler echocardiography as well as color TDI.

## Methods

### Study design and participants

A total of 240 healthy men who were born or permanently live at sea level (SL, < 500 m, above sea level, asl) were recruited in Chongqing, China, and ascended to HA in succession from Yanggongqiao (Chongqing, China, 400 m, asl) to Litang (Sichuan, China, 4100 m, asl) by bus with a stair-like journey within 7 days and finally arrived on July 9, 10, 11 and 12, 2013, respectively. Data collections were performed at SL and within 5 ± 2 h after arriving at 4100 m. Exclusion criteria were known malignant tumors, cardiovascular diseases, any chronic cardiovascular therapy, pulmonary diseases, liver and kidney dysfunction, hematologic diseases, history of HA exposure in the recent 6 months, history of angioedema, and psychiatric disorders. This study was registered under the Chinese Clinical Trial Registration (No: ChiCTR-RCS-12002232, https://www.chictr.org.cn). All procedures and protocols were approved by the Clinical Research Ethics Board at the Army Medical University (Third Military Medical University) (NO: 2012014) and conformed to the standards set by the Declaration of Helsinki. All subjects volunteered to participate in this study and gave written informed consent.

### Clinical examinations

Age, height, body weight and smoking history were recorded. Body mass index (BMI) was calculated as weight (kg)/[height (m)]^2^, body surface area (BSA) was calculated as 0.0061 × height (cm) + 0.0128 × weight (kg) − 0.1529). Resting HR was from electrocardiogram. Systolic blood pressure (SBP) and diastolic blood pressure (DBP) were measured with an Omron HEM-6200 (Japan) with the subject resting in a sitting position for at least 5 min. The oxygen saturation (SaO_2_) values were obtained from warmed hands from the fingertips with a pulse oximeter (Nonin ONYX OR9500, USA) after at least 10 min of rest.

### Echocardiographic image acquisition

Two-dimensional, Doppler and tissue Doppler echocardiography were performed by registered sonographers by using CX50 ultrasound systems (Philips Ultrasound System, Andover, MA, USA) as previously described [13]. All images were obtained at end-expiration with the subjects in the left lateral decubitus position after at least 10 min of rest to allow the subjects to reach steady state. Two-dimensional grayscale harmonic images with 70–90 frames per second and color tissue Doppler images with 110–140 frames per second were saved digitally for subsequent offline analysis (QLAB 10.5; Philips Healthcare, Andover, MA, USA).

### Two-dimensional and Doppler echocardiography

The measurements were conducted according to the current guideline [14]. The LV end-diastolic and end-systolic volumes (EDV and ESV, respectively), stroke volume (SV, = EDV − ESV), cardiac output (CO, = SV × HR) and LV ejection fraction [LVEF, = (EDV − ESV)/EDV × 100] were measured or calculated as previous described (Yang et al. 2015). The early (E), late (A) diastolic wave peak velocities and E/A were measured from mitral or tricuspid pulse-Doppler inflow. According to current guideline [15], the end-systolic and end-diastolic RV areas (ESA and EDA) were obtained on an apical RV focused view, and the fractional area change (FAC) was calculated as follows: (EDA − ESA)/EDA × 100. Pulsed-wave Doppler imaging of the pulmonary artery was performed and the pulmonary acceleration time (AT) and ejection time (ET) were measured, and the mean pulmonary artery pressure (mPAP) was estimated as follows: when the AT was longer than 120 ms, mPAP = 79 − (0.45 × AT), and when the AT was shorter than 120 ms, mPAP = 90 − (0.62 × AT) [6]. The maximal velocity of the tricuspid regurgitation jet (TRV) was measured, and the systolic pulmonary arterial pressure (SPAP) was calculated with the Bernoulli equation (TRV in m/s): SPAP = 4 × TRV^2^ + 10.

### Color tissue Doppler imaging

Tissue velocities were measured from the apical 4-chamber view in a standard manner through color TDI analysis in 147 randomly selected subjects as a subgroup. The systolic (S′) positive wave and early diastolic (E′) tissue velocities were measured at the center of the lateral walls from the mitral or tricuspid annulus from the average of three cardiac cycles (Fig. 2). The E/e′ ratio was calculated to evaluate the LV and RV filling pressures [16]. The myocardial performance index (MPI) was also measured using the TDI method, which allowed a global estimation of both LV and RV systolic and diastolic function [15].

### Reproducibility

Observer reliability of main echocardiographic measurements was assessed in 20 randomly selected subjects. Interobserver variability was performed by two separate observers, and intraobserver variability was performed by the same observer at least 1 month apart. Both the intraobserver and interobserver variabilities were tested using the intraclass correlation coefficient (ICC) by Cronbach’s α.

### Statistical analysis

Statistical analyses were performed using SPSS 22.0 for Windows (IBM Corp., Armonk, NY, USA). Continuous variables were presented as either the mean ± standard deviation (SD) or median (25th, 75th percentile) according to their normality results from the Kolmogorov–Smirnov test. The differences between parameters at sea level and at HA were compared with paired t-tests or Wilcoxon rank–sum tests. Correlations were performed using linear regression. Trend tests were used for cross-group comparisons of continuous variables. Categorical variables were expressed as counts and percentages and analyzed using the χ^2^ test. Statistical power calculations were evaluated (PASS software, version 11, NCSS, LLC, Kaysville, UT, USA), and more than 80% statistical power was achieved for detect significant differences between subgroups using a two-sided alpha of 0.05. All statistical tests were two-sided, and a p-value < 0.05 was statistically significant and was presented in bold in Tables.

## Results

### The general cardiac responses following HA exposure

The basic information of the participants, including age, height, weight, BSA, BMI, ethnicity, and smoking history is shown in Supplemental Table S1. As shown in Table 1, following HA exposure, the HR [65(59, 71) vs. 72(63, 80) beats/min, p < 0.001], SBP and DBP were significantly increased, whereas the SaO_2_ level was decreased compared with the values at SL. The EDVi, and ESVi and the calculated SVi [35.5 (30.5, 42.3) vs. 32.9 (27.4, 39.5) ml/m^2^, p < 0.001] were significantly reduced. Consequently, CI was unaltered. However, the LVEF was improved [(62.1 (56.5, 67.8) vs. 65.2 (58.8, 70.4) %, p = 0.001]. Moreover, there were significant reductions in the mitral peak E-wave velocity, peak A-wave velocity and the E/A ratio. Furthermore, the mitral E′ was significantly reduced (9.0 ± 2.2 vs. 8.2 ± 1.9 cm/s, p < 0.001) but the mitral S′ was comparable before and after HA exposure. Therefore, the LV MPI was significantly increased but the LV filling index (mitral E/E ratio was calculated to evaluate ratio) was also decreased after HA exposure.

**Table 1:** The physiologic parameters and echocardiographic parameters of participants at sea level and at high altitude

Variables	Sea-level	High-altitude	Δ value	P value
Physiologic parameters (n = 240)				
HR (beats/min)	65 (59, 71)	72 (63, 80)	6.4 ± 11.4	** < 0.001**
SaO_2_ (%)	98 (97, 98)	89 (88, 91)	− 8.0 (− 10.0, − 7.0)	** < 0.001**
SBP (mmHg)	112 (106, 119)	120 (113, 127)	8.0 (1.0, 16.0)	** < 0.001**
DBP (mmHg)	67.6 ± 8.6	78.9 ± 9.2	11.3 ± 11.0	** < 0.001**
2D and Doppler echocardiography (n = 240)				
LV EDVi (ml/m^2^)	58.2 (50.2, 66.6)	51.7 (43.6, 59.0)	− 6.8 (− 16.6, 3.0)	** < 0.001**
LV ESVi (ml/m^2^)	22.5 ± 7.9	18.4 ± 5.8	− 4.0 ± 7.7	** < 0.001**
SVi (ml/m^2^)	35.5 (30.5, 42.3)	32.9 (27.4, 39.5)	− 2.7 (− 9.6, 3.8)	** < 0.001**
CI (L/min/m^2^)	2.2 (1.9, 2.7)	2.3 (1.8, 2.8)	0.05 (− 0.53, 0.56)	0.652
LVEF (%)	62.1 (56.5, 67.8)	65.2 (58.8, 70.4)	2.5 (− 5.0, 9.0)	**0.001**
Mitral E (cm/s)	98.1 (88.6, 107.2)	78.7 (68.0, 91.8)	− 17.1 ± 18.0	** < 0.001**
Mitral A (cm/s)	51.7 ± 11.7	49.6 ± 10.8	− 1.1 ± 15.0	**0.034**
Mitral E/A ratio	1.90 (1.57, 2.27)	1.61 (1.36, 1.92)	− 0.26 (− 0.63, 0.07)	** < 0.001**
RV EDAi (cm^2^/m^2^)	13.4 (12.1, 15.0)	12.3 (11.1, 14.2)	− 1.2 ± 2.8	** < 0.001**
RV ESAi (cm^2^/m^2^)	7.5 (6.6, 8.2)	7.1 (6.3, 8.4)	− 0.2 ± 1.7	0.183
RV FAC (%)	45.5 (42.3, 48.0)	41.8 (38.0, 44.8)	− 3.5 ± 5.4	** < 0.001**
Tricuspid E (cm/s)	74.1 ± 13.0	61.9 ± 12.7	− 12.2 ± 15.0	** < 0.001**
Tricuspid A (cm/s)	36.0 (30.6, 42.2)	33.1 (28.4, 40.0)	− 4.1 (− 10.5, 3.6)	** < 0.001**
Tricuspid E/A ratio	2.00 (1.67, 2.38)	1.80 (1.46, 2.25)	− 0.18 ± 0.68	** < 0.001**
Color tissue doppler imaging (n = 147)				
Mitral S′ (cm/s)	6.3 ± 1.6	6.2 ± 1.6	− 0.1 ± 2.2	0.416
Mitral E′ (cm/s)	9.0 ± 2.2	8.2 ± 1.9	− 0.8 ± 2.5	** < 0.001**
Mitral E/E′ ratio	10.6 (9.3, 12.7)	9.2 (7.8, 11.9)	− 1.2 (− 3.6, 1.2)	** < 0.001**
LV MPI	0.41 ± 0.13	0.48 ± 0.15	0.06 ± 0.19	** < 0.001**
Tricuspid S′ (cm/s)	7.7 ± 1.5	7.6 ± 1.3	− 0.2 ± 1.7	0.255
Tricuspid E′ (cm/s)	7.2 (5.7, 8.6)	7.5 (6.0, 8.9)	0.3 (− 1.2, 1.9)	0.186
Tricuspid E/E′ ratio	10.4 (8.5, 13.0)	8.5 (6.7, 10.4)	− 1.8 (− 4.6, 0.2)	** < 0.001**
RV MPI	0.52 (0.38, 0.67)	0.63 (0.48, 0.75)	0.09 ± 0.28	** < 0.001**
Pulmonary haemodynamics (n = 240)				
AT/ET	0.37 (0.34, 0.40)	0.31 (0.21, 0.35)	− 0.06 ± 0.07	** < 0.001**
mPAP (mmHg)	18.6 (15.4, 22.3)	24.3 (20.2, 32.6)	7.7 (1.1, 15.6)	** < 0.001**
TR [n (%)]	135 (56.3)	192 (80.0)		** < 0.001**
TRV (m/s)	2.15 (1.91, 2.33)	2.46 (2.26, 2.78)		** < 0.001**
SPAP (mmHg)	28.5 (24.7, 31.8)	34.1 (30.4, 41.0)		** < 0.001**

Values are median (25th to 75th quartile) or mean ± SD

*A* mitral or tricuspid inflow late diastolic velocity; *AT* pulmonary acceleration time; *CI* cardiac index; *DBP* diastolic blood pressure; *E* mitral or tricuspid inflow early diastolic velocity; *ET* pulmonary ejection time; *E*′ peak early diastolic velocity with tissue Doppler imaging at the mitral or tricuspid annular; *FAC* right ventricular fractional area of change; *HR* heart rate; *LV* left ventricular; *LV EDVi* left ventricular end-diastolic volume index; *LV ESVi* left ventricular end-systolic volume index; *LVEF* left ventricle ejection fraction; *MPI* myocardial performance index; *RV* right ventricular; *RV EDAi* right ventricular end-diastolic area index; *RV ESAi* right ventricular end-systolic area index; *S*′ peak systolic velocity with tissue Doppler imaging at the mitral or tricuspid annular; *E*′ early diastolic velocity at the mitral or tricuspid annular; *SaO*_*2*_ Oxygen saturation; *SBP* systolic blood pressure; *SPAP* systolic pulmonary artery pressure; *SVi* stroke volume index; *TR* tricuspid regurgitation; *TRV* tricuspid regurgitation velocity

Following HA exposure, the decrease in RV EDAi and the unchanged RV ESAi resulted in a significant reduction in RV FAC [45.5 (42.3, 48.0) vs. 41.8 (38.0, 44.8) %, p < 0.001]. Furthermore, there were significant reductions in tricuspid peak E-wave velocity, peak A-wave velocity and the E/A ratio, although the tricuspid S′ and tricuspid E′ remained unchanged. Consequently, the tricuspid E/E′ ratio was decreased. Nevertheless, the RV MPI and mPAP were significantly increased, and the percentage of subjects with functional TR increased from 56.3% to 80.0%. The calculated SPAP from TRV was also increased (Table 1).

### Associations of baseline HR and the changes in HR and CI values following HA exposure

**Fig. 1:**
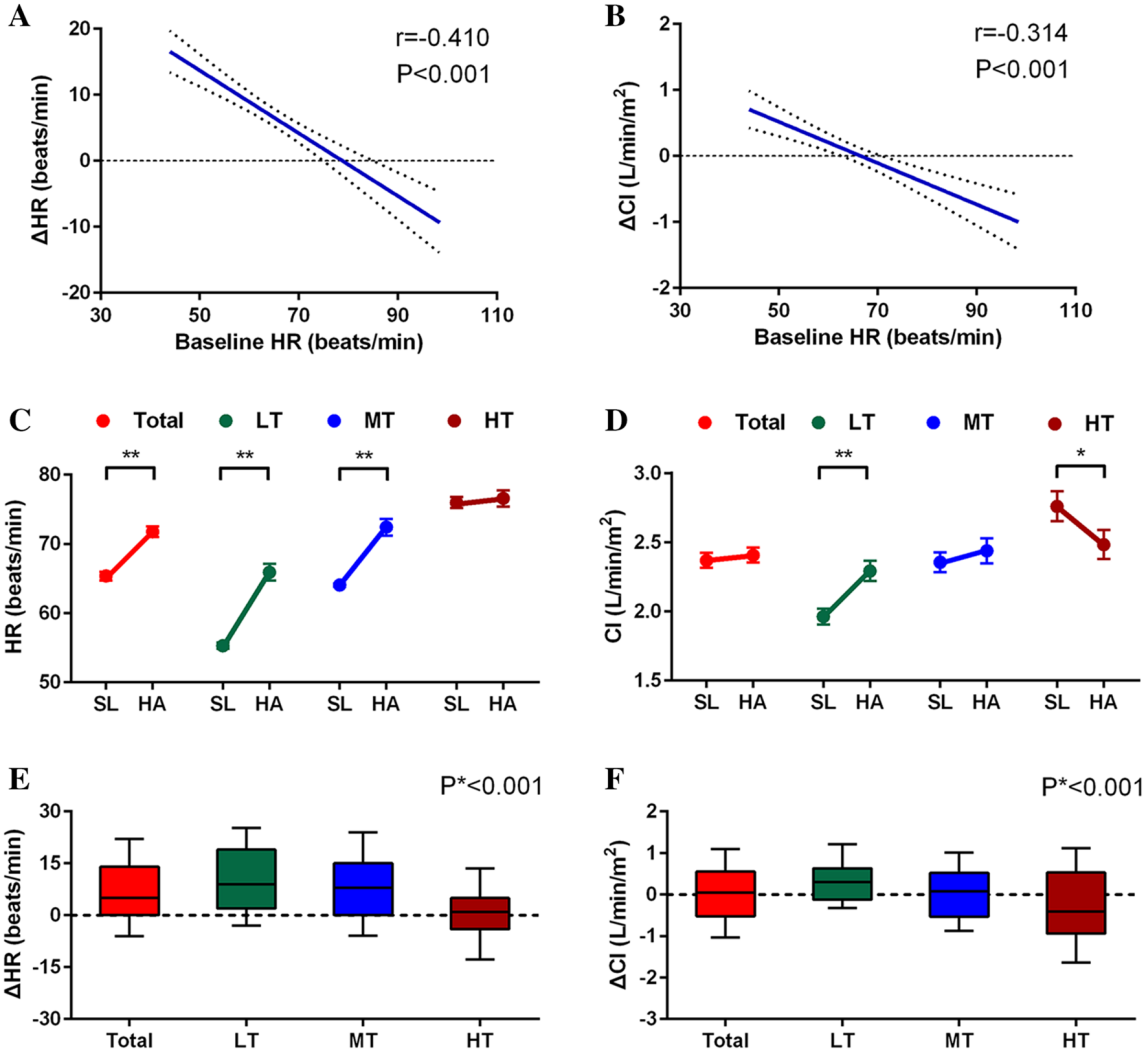
Correlations of baseline HR with the changes in HR and CI in response to HA exposure. The change in the values (Δvalues) of HR (**a**) and CI (**b**) were negatively correlated with the baseline HR after HA exposure. The effects of HA exposure on HR (**c**), the CI (**d**), and their Δvalues (**e**, **f**) in total subjects and different tertiles of baseline HR. *SL* sea level, *HA* high altitude, *HR* heart rate, *CI* cardiac index, *LT* lowest tertile HR, *MT* middle tertile HR, *HT* highest tertile HR, *p < 0.05, **p < 0.01, p*: p value for trend

Results from the linear regression analysis identified that the **Δ**HR after HA exposure were negatively associated with the baseline HR (r = − 0.410, p < 0.001) (Fig. 1a). Moreover, the ΔCI was also negatively associated with the baseline HR (r = − 0.314, p < 0.001) (Fig. 1b).

### LV functional responses to HA exposure in subjects with different tertiles of baseline HR

The subjects in our study were divided into three groups based on the tertiles of their baseline HR at SL: lowest tertile HR (LT), middle tertile HR (MT) and highest tertile HR (HT), and their baseline characteristics were summarized in Supplemental Table S1, which showed no significant differences. However, following HA exposure, the HR was significantly increased in the LT [56 (53, 58) vs. 65 (58, 73) beats/min, p < 0.001] and MT groups but not in the HT group; the **Δ**HR was higher in the LT group than in the MT group (Fig. 1c, e), although the changes in SBP, DBP and SaO_2_ value were equally among the groups (Table 2).

**Table 2:** Physiologic parameters and Left ventricular parameters of participants in different baseline resting heart rate at sea level and at high altitude

Variables	Lowest tertile		P value	Middle Tertile		P value	Highest tertile		P value
	Sea level	High altitude		Sea level	High altitude		Sea level	High altitude	
Physiologic parameters (n = 77/80/83)									
HR (beats/min)	56 (53, 58)	65 (58, 73)	** < 0.001**	64 (62, 66)	72 (64, 78)	** < 0.001**	75 (70, 79)	77 (69, 84)	0.280
SaO_2_ (%)	98 (97, 98)	89 (88, 91)	** < 0.001**	98 (97, 98)	89 (88, 91)	** < 0.001**	98 (97, 98)	89 (88, 91)	** < 0.001**
SBP (mmHg)	112 (107, 119)	120 (112, 127)	** < 0.001**	110 (104, 118)	118 (112, 126)	** < 0.001**	115 (108, 120)	123 (115, 127)	** < 0.001**
DBP (mmHg)	67.0 ± 8.5	78.9 ± 8.8	** < 0.001**	67.3 ± 9.0	77.4 ± 8.9	** < 0.001**	68.4 ± 8.2	80.5 ± 9.6	** < 0.001**
2D and Doppler echocardiography (n = 77/80/83)									
LV EDVi (ml/m^2^)	57.1 (50.2, 66.3)	54.2 (44.8, 58.9)	**0.004**	58.1 (51.1, 65.8)	52.2 (45.4, 59.6)	** < 0.001**	58.7 (49.2, 67.8)	49.1 (41.5, 58.9)	** < 0.001**
LV ESVi (ml/m^2^)	22.2 ± 7.6	18.4 ± 5.8	** < 0.001**	22.5 ± 7.1	18.9 ± 5.9	** < 0.001**	22.6 ± 8.8	18.0 ± 5.7	** < 0.001**
SVI (ml/m^2^)	35.9 (30.6, 41.2)	33.5 (29.1, 39.9)	0.268	35.8 (30.8, 41.5)	33.2 (28.1, 39.8)	**0.012**	33.6 (30.0, 44.1)	30.6 (23.8, 39.0)	**0.004**
CI (L/min/m^2^)	1.9 (1.6, 2.3)	2.2 (1.8, 2.6)	** < 0.001**	2.3 (2.0, 2.6)	2.3 (1.9, 2.8)	0.568	2.5 (2.1, 3.4)	2.3 (1.8, 3.1)	**0.012**
LVEF (%)	61.7 (56.5, 68.0)	66.1 (60.7, 71.5)	**0.004**	61.6 (56.1, 67.5)	64.6 (58.5, 71.5)	0.128	63.1 (55.7, 69.1)	64.9 (57.5, 69.3)	0.112
Mitral E (cm/s)	99.9 (90.5, 111.8)	75.2 (68.0, 90.0)	** < 0.001**	96.6 (88.4, 105.4)	79.5 (68.9, 91.6)	** < 0.001**	99.9 (90.5, 111.8)	75.2 (68.0, 90.0)	** < 0.001**
Mitral A (cm/s)	48.0 ± 11.2	46.2 ± 10.1	0.324	50.4 ± 11.1	51.2 ± 11.9	0.662	56.4 ± 11.3	51.3 ± 9.8	** < 0.001**
Mitral E/A ratio	2.16 (1.71, 2.50)	1.71 (1.47, 2.15)	** < 0.001**	1.89 (1.65, 2.27)	1.61 (1.31, 1.86)	** < 0.001**	1.78 (1.45, 2.07)	1.53 (1.33, 1.81)	** < 0.001**
Color tissue doppler imaging (n = 43/47/57)									
Mitral S′ (cm/s)	5.8 ± 1.4	6.5 ± 1.9	**0.040**	5.8 ± 1.6	6.5 ± 1.5	**0.035**	6.7 ± 1.8	6.3 ± 1.5	0.167
Mitral E′ (cm/s)	8.7 ± 2.6	8.2 ± 1.9	0.262	8.9 ± 2.0	8.0 ± 2.0	**0.008**	9.2 ± 2.0	8.4 ± 1.8	**0.003**
Mitral E/E′ ratio	10.6 (8.6, 13.6)	8.6 (7.6, 12.4)	0.058	10.7 (9.4, 12.6)	9.5 (8.6, 11.6)	**0.014**	10.3 (9.2, 12.0)	8.7 (7.3, 11.2)	**0.011**
LV MPI	0.42 ± 0.16	0.48 ± 0.17	0.097	0.44 ± 0.12	0.51 ± 0.16	**0.014**	0.38 ± 0.12	0.45 ± 0.14	**0.001**

Values are median (25th to 75th quartile) or mean ± SD

Abbreviations as in Table 1

**Fig. 2:**
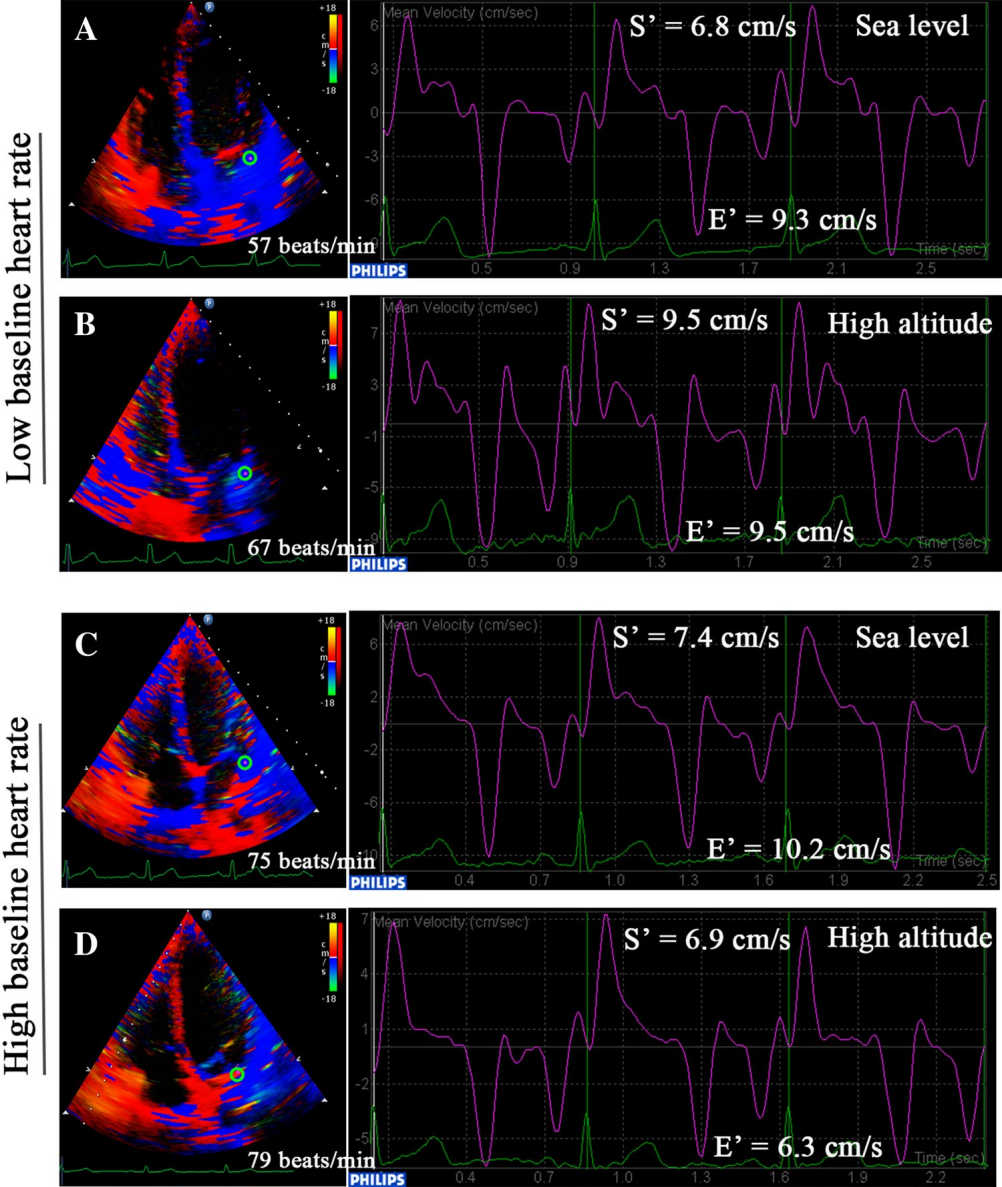
Color tissue Doppler imaging and the curves. The systolic (S′) positive wave and early diastolic (E′) tissue velocities were measured at the center of the lateral walls from the mitral annulus in subjects with lowest tertile baseline HR at sea level (**a**) and high altitude (**b**), with highest tertile baseline HR at sea level (**c**) and high altitude (**d**)

**Fig. 3:**
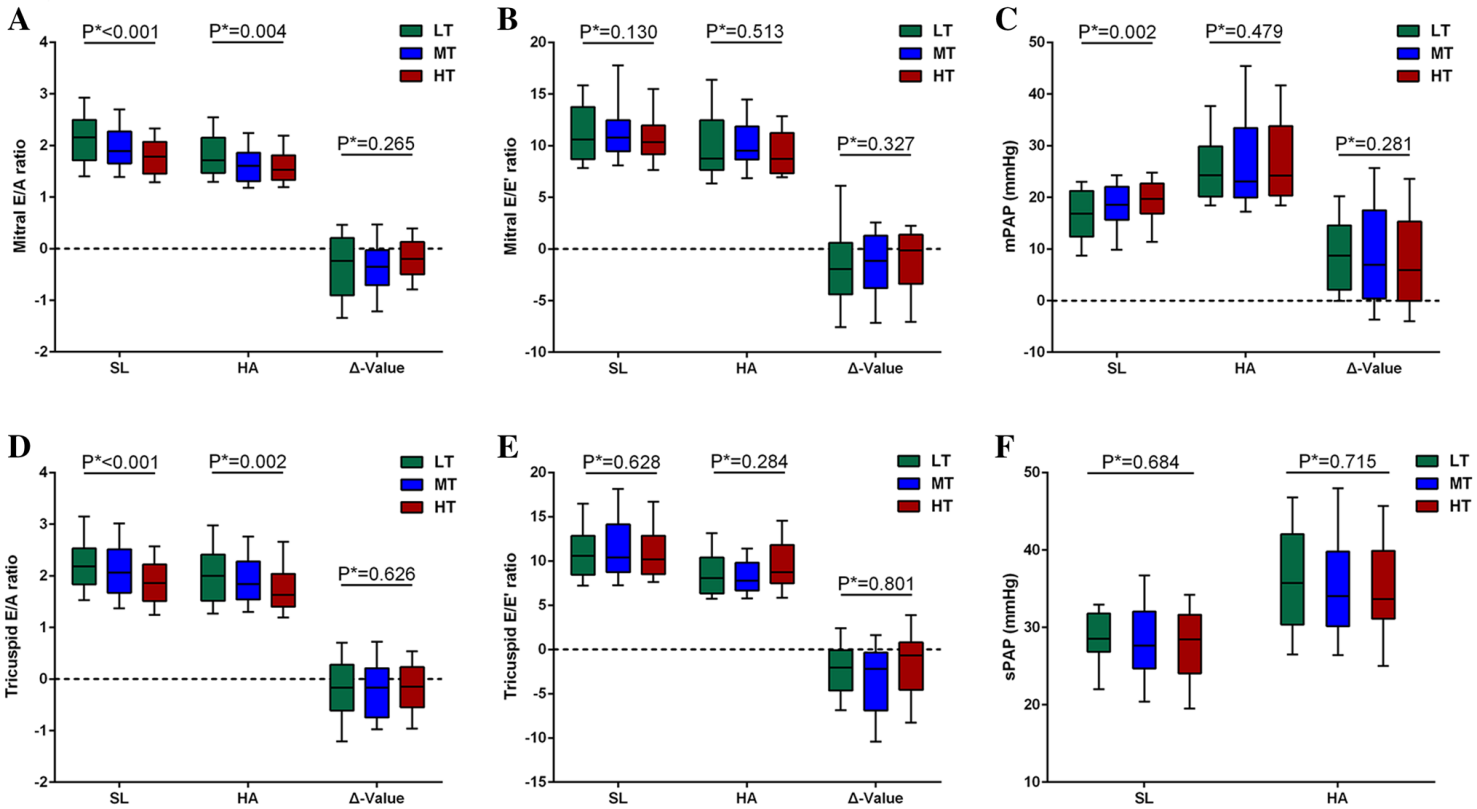
The effects of different baseline HRs on ventricular fillings and RV afterload in subjects at SL or HA. **a** Mitral E/A ratio, **b** mitral E/e′ ratio, **c** mean pulmonary artery pressure (mPAP), **d** tricuspid E/A ratio, **e** tricuspid E/e′ ratio, **f** systolic pulmonary artery pressure (SPAP). p*: p value for trend

After HA exposure, the EDVi and ESVi uniformly decreased in all three groups; no significant differences were found in **Δ**EDVi and **Δ**ESVi, although the EDVi and ESVi for subjects at SL gradually decreased with increasing baseline HR. However, the calculated SVi, which was decreased in total subjects, was unchanged in the LT group but decreased in the MT and HT groups, and the CI increased in the LT group but decreased in the HT group (Fig. 1d, f). Moreover, the LVEF only significantly increased in the LT group [61.7 (56.5, 68.0) vs. 66.1 (60.7, 71.5) %, p = 0.004] but not in the MT and HT groups. Although the mitral E/A ratios of subjects at SL gradually decreased with increasing baseline HR, the mitral peak E-wave and the E/A ratio uniformly decreased in all three groups after HA exposure (Fig. 3a). Interestingly, the mitral S′ significantly increased in the LT group (5.8 ± 1.4 vs. 6.5 ± 1.9 cm/s, p = 0.040) (Fig. 2a, b), whereas the mitral E′ was decreased in the MT (8.9 ± 2.0 vs. 8.0 ± 2.0 cm/s, p = 0.008) and HT (9.2 ± 2.0 vs. 8.4 ± 1.8 cm/s, p = 0.003) groups (Fig. 2c, d); the mitral E/E′ ratio and LV MPI were decreased in the MT and HT groups but not in the LT group following HA exposure (Table 2).

### RV functional responses to HA exposure in subjects with different tertiles of baseline HR

As shown in Table 3, after HA exposure, the RV EDAi and the calculated RV FAC uniformly decreased in all three groups. Similarly, subjects in all the three groups showed uniform reductions in E-wave velocity, E/A, and E/E′ and increases in the mPAP, TR, TRV and SPAP, although the mitral and tricuspid E/A ratio of subjects at SL gradually decreased with increasing baseline HR (Fig. 3). Moreover, no significant differences were found in the tricuspid S′ and E′ among the three groups. However, increases in the RV MPI were found in the LT and HT groups but not in the MT group.

**Table 3:** Right ventricular parameters of participants in different baseline resting heart rate at sea level and at high altitude

Variables	Lowest tertile		P value	Middle tertile		P value	Highest tertile		P value
	Low-altitude	High-altitude		Low-altitude	High-altitude		Low-altitude	High-altitude	
2D and Doppler echocardiography (n = 77/80/83)									
RV EDAi (cm^2^/m^2^)	13.6 (12.2, 15.0)	12.2 (11.1, 14.0)	** < 0.001**	14.1 (12.7, 15.3)	13.1 (11.2, 15.1)	**0.007**	12.8 (11.2, 14.9)	12.0 (10.9, 13.7)	**0.016**
RV ESAi (cm^2^/m^2^)	7.7 (6.9, 8.3)	7.0 (6.6, 8.4)	**0.036**	7.4 (6.7, 8.6)	7.9 (6.4, 8.8)	0.988	7.2 (6.0, 8.1)	7.0 (5.8, 8.0)	0.602
RV FAC (%)	44.0 (42.1, 48.2)	42.0 (38.6, 44.0)	** < 0.001**	45.9 (42.7, 48.8)	40.6 (37.5, 44.2)	** < 0.001**	45.5 (41.9, 47.8)	42.0 (39.1, 45.5)	** < 0.001**
Tricuspid E (cm/s)	75.4 ± 11.1	62.4 ± 13.4	** < 0.001**	73.4 ± 12.0	61.5 ± 12.0	** < 0.001**	73.6 ± 15.2	61.8 ± 12.9	** < 0.001**
Tricuspid A (cm/s)	34.3 (29.3, 41.3)	31.0 (24.9, 39.0)	0.100	35.5 (29.7, 39.5)	31.1 (27.7, 37.3)	**0.018**	38.5 (33.1, 48.3)	35.7 (31.4, 41.7)	**0.006**
Tricuspid E/A ratio	2.18 (1.84, 2.53)	2.00 (1.52, 2.42)	**0.038**	2.07 (1.67, 2.52)	1.84 (1.54, 2.28)	**0.016**	1.86 (1.51, 2.21)	1.62 (1.40, 2.02)	**0.032**
Color tissue doppler imaging (n = 43/47/57)									
Tricuspid S′ (cm/s)	7.9 ± 1.2	7.7 ± 1.4	0.515	7.7 ± 1.7	7.7 ± 1.2	0.990	7.6 ± 1.6	7.3 ± 1.4	0.169
Tricuspid E′ (cm/s)	7.1 (5.7, 8.4)	7.6 (5.8, 9.1)	0.284	7.1 (5.4, 8.7)	7.8 (6.5, 9.0)	0.105	7.3 (5.2, 8.4)	7.2 (5.6, 8.8)	0.646
Tricuspid E/E′ ratio	10.6 (8.4, 12.8)	8.1 (6.3, 10.4)	**0.009**	10.4 (8.7, 14.2)	7.8 (6.7, 9.8)	** < 0.001**	10.1 (8.5, 12.9)	8.8 (7.4, 12.0)	**0.034**
RV MPI	0.53 (0.40, 0.67)	0.63 (0.52, 0.81)	**0.004**	0.58 (0.47, 0.74)	0.64 (0.48, 0.75)	0.342	0.49 (0.33, 0.63)	0.63 (0.45, 0.75)	**0.004**
Pulmonary haemodynamics (n = 77/80/83)									
AT/ET	0.38 (0.34, 0.40)	0.31 (0.28, 0.35)	** < 0.001**	0.37 (0.34, 0.41)	0.31 (0.26, 0.35)	** < 0.001**	0.37 (0.35, 0.40)	0.32 (0.27, 0.35)	** < 0.001**
mPAP, mmHg	16.9 (12.4, 21.3)	24.3 (20.1, 29.9)	** < 0.001**	18.6 (15.6, 22.1)	23.1 (20.0, 33.5)	** < 0.001**	19.7 (16.9, 22.7)	24.2 (20.4, 33.8)	** < 0.001**
TR [n (%)]	38 (49.4)	61 (79.2)	** < 0.001**	48 (60.0)	68 (85.0)	** < 0.001**	49 (59.0)	63 (75.9)	**0.020**
TRV (m/s)	2.15 (2.05, 2.33)	2.54 (2.26, 2.83)	** < 0.001**	2.10 (1.92, 2.35)	2.45 (2.24, 2.73)	** < 0.001**	2.15 (1.87, 2.33)	2.43 (2.30, 2.73)	** < 0.001**
SPAP (mmHg)	28.5 (26.9, 31.8)	35.7 (30.4, 42.1)	** < 0.001**	27.7 (24.7, 32.1)	34.0 (30.1, 39.8)	** < 0.001**	28.4 (24.0, 31.6)	33.7 (31.1, 39.9)	** < 0.001**

Values are median (25th to 75th quartile) or mean ± SD

Abbreviations as in Table 1

### Inter- and intra-observer variability

The intraclass correlation coefficients for absolute agreement (ICCa) of the main cardiac structural and functional parameters for the intra- and inter-observer measurements are shown in Supplemental Table S2. All the measurements showed excellent or good agreements.

## Discussion

**Fig. 4:**
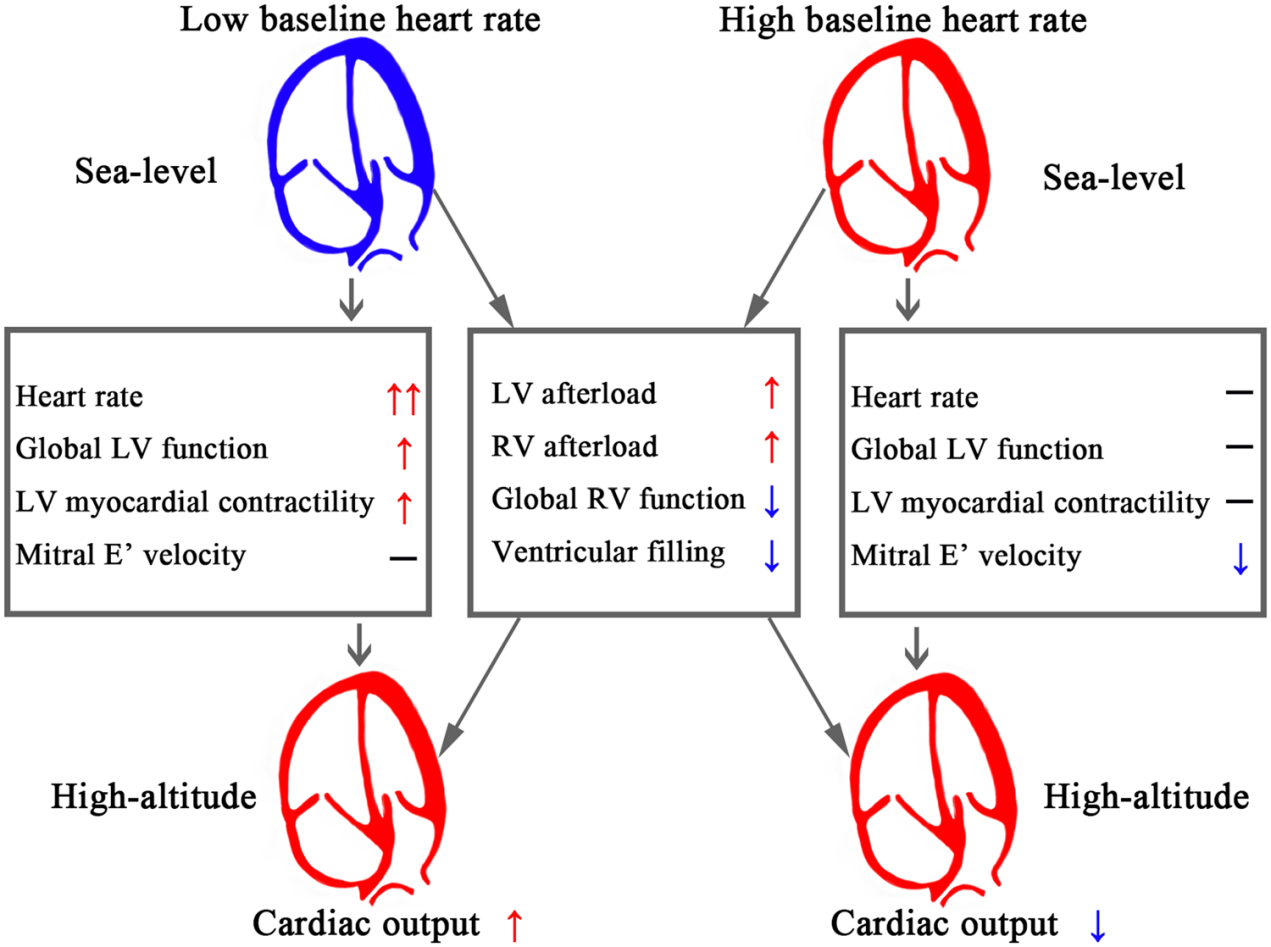
Central Illustration. The figure shows that there was good adaptation in subjects with a low baseline HR and that there was a reduced LV myocardial velocity in early diastole in subjects with a high baseline HR. *LV* left ventricular, *RV* right ventricular

In this study, we demonstrated that the baseline HR of subjects at SL was associated with the changes in HR and CI after HA exposure. The HR increased in the LT and MT groups but not in the HT group, and the SVi decreased in the MT and HT groups but not in the LT group; this resulted in an increase in CI in the LT group, an unchanged CI in the MT group and a decreased CI in the HT group. Furthermore, after acute HA exposure, the global LV systolic function and myocardial contractility were enhanced, and the LV myocardial velocity in early diastole was unchanged for subjects in the LT group; however, while the global LV systolic function and myocardial contractility were unaltered, there was a decline in LV myocardial velocity in early diastole for subjects in the HT group. Our findings indicated that the baseline HR of subjects at sea level could determine the cardiac responses to acute HA exposure, which are characterized by enhanced LV function in subjects with a low baseline HR and by reduced LV myocardial velocity in early diastole in subjects with a high baseline HR (Fig. 4).

The reasons for the decline in SVi have been continuously debated for over 40 years, but the exact mechanisms involved are still not completely understood. Undoubtedly, HR, preload, afterload and ventricular function are the main determinants of SV [9]. In this study, we found that an increase in HR was accompanied by reductions in the mitral and tricuspid E/A inflow ratios and E/e′ ratio, whereas a high HR at HA could shorten the diastolic period, which then reduces LV filling [17]. Moreover, we also found that the mPAP was greatly elevated and the RV function decreased. During acute HA exposure, the high mPAP was mainly attributed to HPV, which in turn increased the RV afterload, impaired RV function and led to ventricular mismatch. Subsequently, these alterations decreased the volume of blood returning to the left ventricle, thus lowering the SV [18]. In our study, the abovementioned factors that potentially affect SV might be compensated by an enhanced LV systolic function, which results from the unchanged SVi in subjects with a low baseline HR. Additionally, in our present study, the decrease in SVi following acute HA exposure for subjects with a high baseline HR might be attributed to a combination of the abovementioned factors with the unaltered LV systolic function as well as impaired LV myocardial relaxation; this parameter has been suggested to be an important factor for the decrease in SV and was associated with the decrease in cardiac high-energy phosphate metabolism under acute HA hypoxia [19].

Due to the shortening of the diastolic period, subjects with a high baseline HR showed lower ventricular filling at sea level than at HA, as described in the present study and by previous data [20]. However, following acute HA exposure, the changes in ventricular filling seemed to be equal across subjects with different levels of baseline HR. On the other hand, the increases in the afterload of both ventricles, which could also contribute to the decline in SV, were also uniform across subjects with different levels of baseline HRs. These uniform changes suggest that there must be other reasons that account for the diverse changes in SV for subjects with different levels of baseline HRs after acute HA exposure. The increase in HR was more pronounced in subjects with a low baseline HR than in those with a high baseline HR, which might be attributed to the progressive decrease in maximal heart rate or heart rate reserve with increasing hypoxia [21]; however, the LV myocardial systolic velocity (S′) also increased in these low baseline HR subjects following acute HA exposure, which led to an increased LVEF, and consequently, the SVi was maintained. Moreover, the resting heart rate was positively correlated with LV S′ as well as E′, independent of sex and age [22]. In our study, although the LV E′ at HA was unchanged in subjects in the LT group, subjects in the MT and HT group showed a significant decrease in LV E′, which suggested that a high baseline HR was associated with impaired LV myocardial relaxation, restoring forces, or increased lengthening load under acute HA exposure and might additionally contribute to the decrease in SV in these subjects [23]. Similarly, by reducing coronary perfusion and increasing myocardial O_2_ consumption during HA exposure, the elevated HR observed in patients with metabolic syndromes resulted in a deterioration of LV function via impaired LV filling and relaxation [24]. In contrast, pure HR reduction by ivabradine, a selective inhibitor of the pacemaker I(f) current, could prevent cardiac dysfunction and fibrosis [25]. Indeed, the autonomic nervous system response was upregulated under acute HA hypoxia, and the normal HR response characterized by HR acceleration from baseline acted as a compensatory mechanism to meet the body's increasing need for O_2_; however, an abnormal HR response in patients with nonobstructive coronary artery disease was usually associated with impaired cardiac function [26].

HR, which is the most important determinant of myocardial oxygen consumption, plays a central role in the cardiac adaptation to sources of metabolic stress, such as hypoxia. The results from the Framingham heart study demonstrated that all-cause mortality increased by 14% for every increase of 10 bpm in the baseline HR, and subjects with a baseline HR > 80 bpm were significantly associated with a high risk of heart failure [27]. In addition, increasing evidence has documented that a low baseline HR usually represents a relatively good clinical outcome, and vice versa. Therefore, the baseline HR not only acted as a physiological predicator of the deterioration of cardiac function but also acted as a prognostic indicator of cardiovascular events [28]. In the present study, although the altered ventricular fillings, the elevated LV and RV afterload and the impairment of RV function might equally contribute to hypoxic adaptation in subjects with different baseline HRs, we also found that subjects with a low baseline HR adapted well to acute HA hypoxia by effectively utilizing heart rate reserve and enhancing LV myocardial contractility; in contrast, subjects with a high baseline HR showed a restricted HR response as well as reduced LV myocardial velocity in early diastole in response to acute HA exposure, which may be because a high baseline HR was associated with exaggerated energy expenditure, impaired myocardial oxygen delivery due to a short diastole period, and loss of the positive force–frequency relationship (Bowditch effect) [29]. Although these reasons could lead to a decrease in SV for subjects with a high baseline HR, the decreased SV and restricted HR could result in a reduced CO, which was suggested to be a potential stimulus for cardiac functional remodeling if the HA exposure was prolonged [30].

## Study limitations

The present observational study has some limitations. First, the enrolled participants were healthy young men, and whether the established results could be generalized to other types of individuals or circumstances (such as women, old adults, children, ascent to HA by other transport modes) is still unknown. Second, due to its complex anatomy, imaging the right ventricle with echocardiography is not an ideal approach. However, the evaluations of the right ventricle in our study were based on the guidelines published by the American Society of Echocardiography [31], and magnetic resonance imaging to assess the right ventricle is not feasible in a field study at HA. Finally, 2-dimensional echocardiography, like other imaging tools, has inherent limitations that impede a thorough evaluation of complex 3-dimensional anatomy. However, it is not ethical or practical to perform right heart catheterization in healthy individuals at HA for research purposes only. Nevertheless, echocardiography remains a simple and feasible method for field studies of HA. In the present study, the same well-trained operators who adopted strict reading criteria and were blinded to the subjects’ grouping information performed all examinations. Therefore, such an approach could reduce the abovementioned limitations to a high degree.

## Conclusion

For the first time, we demonstrated that the baseline HR of subjects at sea level could determine the cardiac responses to acute HA exposure, which were characterized by enhanced LV function in subjects with a low baseline HR and by reduced LV myocardial velocity in early diastole in subjects with a high baseline HR. Our findings provide novel insights into the cardiac responses to acute HA exposure, which could improve our understanding of the cardiac adaptations to acute HA hypoxia and guide our decision making for HA travels or work. Further investigations need to be performed to identify these effects in a larger population or in other types of subjects, such as patients with cardiovascular diseases.

## Electronic supplementary material

Below is the link to the electronic supplementary material.

Supplementary file1 (DOC 47 kb)

Supplementary file2 (DOC 59 kb)
