# Supplementary material for: Effects of baseline heart rate at sea level on cardiac responses to high-altitude exposure
Source: Int J Cardiovasc Imaging. 2020 Jan 17;36(5):799–810. doi: 10.1007/s10554-020-01769-w (PMC7174267; doi:10.1007/s10554-020-01769-w)
Supplement: Supplementary file 1 — Supplementary file1 (DOC 47 kb) [file 10554_2020_1769_MOESM1_ESM.doc]

**Supplemental Table 1.** **Basic characteristics of participants in different baseline resting heart rate.**

| **Variables** | **Total**  **(n=240)** | **Lowest Tertile**  **HR (44-60, n=77)** | **Middle Tertile**  **HR (61-67, n=80)** | **Highest Tertile**  **HR(68-99, n=83)** |
| --- | --- | --- | --- | --- |
| Age, year | 20 (19 - 22) | 21 (19 - 23) | 20 (19 - 22) | 20 (19 - 21) |
| Height, cm | 172.1 ± 4.4 | 172.4 ± 4.6 | 171.7 ± 4.5 | 172.1 ± 3.9 |
| Weight, kg | 63.1 ± 6.6 | 64.3 ± 6.7 | 62.2 ± 6.4 | 62.9 ± 6.5 |
| Body surface area, m^2^ | 1.71 ± 0.10 | 1.72 ± 0.10 | 1.69 ± 0.10 | 1.71 ± 0.10 |
| Body mass index, kg/m^2^ | 21.0 (19.8 - 22.7) | 21.2 (19.8 - 23.1) | 20.8 (19.8 - 22.5) | 21.2 (19.8 - 22.3) |
| Han ethnic, n (%) | 208 (86.7) | 63 (81.8) | 71 (88.8) | 74 (89.2) |
| Smoking status, n (%) | 166 (69.2) | 55 (71.4) | 52 (65.0) | 59 (71.1) |

Values are median (25th to 75th quartile) or n (%).
