# Supplementary material for: Effects of baseline heart rate at sea level on cardiac responses to high-altitude exposure
Source: Int J Cardiovasc Imaging. 2020 Jan 17;36(5):799–810. doi: 10.1007/s10554-020-01769-w (PMC7174267; doi:10.1007/s10554-020-01769-w)
Supplement: Supplementary file 2 — Supplementary file2 (DOC 59 kb) [file 10554_2020_1769_MOESM2_ESM.doc]

**Supplemental Table 2**. **ICC analysis of** **intra- and interobserver variations.**

| **Variables** | **Intraobserver variation** | | **P value** | **Interobserver variation** | | **P value** |
| --- | --- | --- | --- | --- | --- | --- |
|  | ICC | 95%CI |  | ICC | 95%CI |  |
| Mitral E, cm/s | 0.990 | 0.975-0.996 | <0.001 | 0.982 | 0.956-0.993 | <0.001 |
| Mitral A, cm/s | 0.983 | 0.958-0.993 | <0.001 | 0.979 | 0.948-0.992 | <0.001 |
| Mitral S’, cm/s | 0.984 | 0.961-0.994 | <0.001 | 0.975 | 0.937-0.990 | <0.001 |
| Mitral E’, cm/s | 0.975 | 0.937-0.990 | <0.001 | 0.883 | 0.705-0.954 | <0.001 |
| LV MPI | 0.929 | 0.828-0.971 | <0.001 | 0.881 | 0.707-0.952 | <0.001 |
| RVEDA, cm^2^ | 0.986 | 0.965-0.994 | <0.001 | 0.993 | 0.982-0.997 | <0.001 |
| RVESA, cm^2^ | 0.978 | 0.944-0.991 | <0.001 | 0.972 | 0.928-0.989 | <0.001 |
| AT, ms | 0.873 | 0.685-0.949 | <0.001 | 0.987 | 0.967-0.995 | <0.001 |
| ET, ms | 0.967 | 0.918-0.987 | <0.001 | 0.974 | 0.935-0.990 | <0.001 |
| TRV, cm/s | 0.969 | 0.924-0.988 | <0.001 | 0.971 | 0.927-0.989 | <0.001 |
| Tricuspid E, cm/s | 0.989 | 0.972-0.996 | <0.001 | 0.967 | 0.918-0.987 | <0.001 |
| Tricuspid A, cm/s | 0.961 | 0.903-0.984 | <0.001 | 0.954 | 0.885-0.982 | <0.001 |
| Tricuspid S’, cm/s | 0.972 | 0.931-0.989 | <0.001 | 0.944 | 0.858-0.978 | <0.001 |
| Tricuspid E’, cm/s | 0.910 | 0.778-0.964 | <0.001 | 0.878 | 0.691-0.952 | <0.001 |
| RV MPI | 0.954 | 0.889-0.981 | <0.001 | 0.946 | 0.864-0.979 | <0.001 |

Abbreviations as in Table 1.
